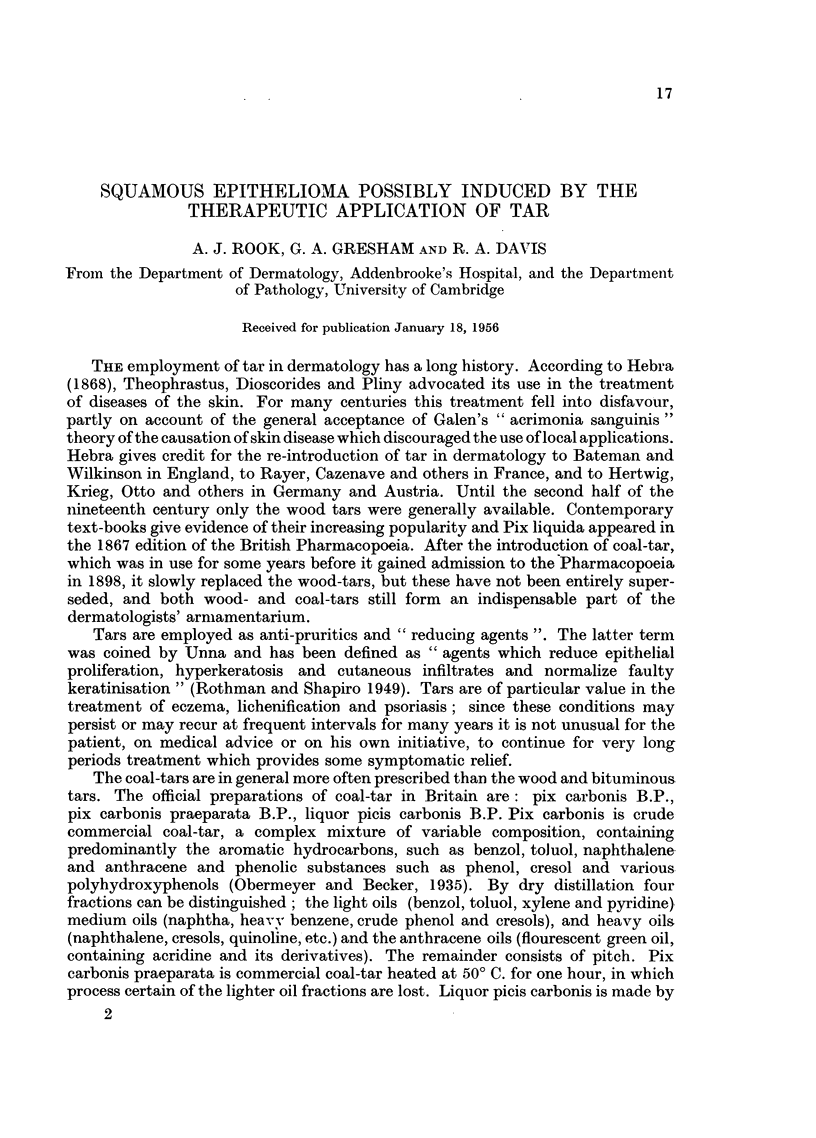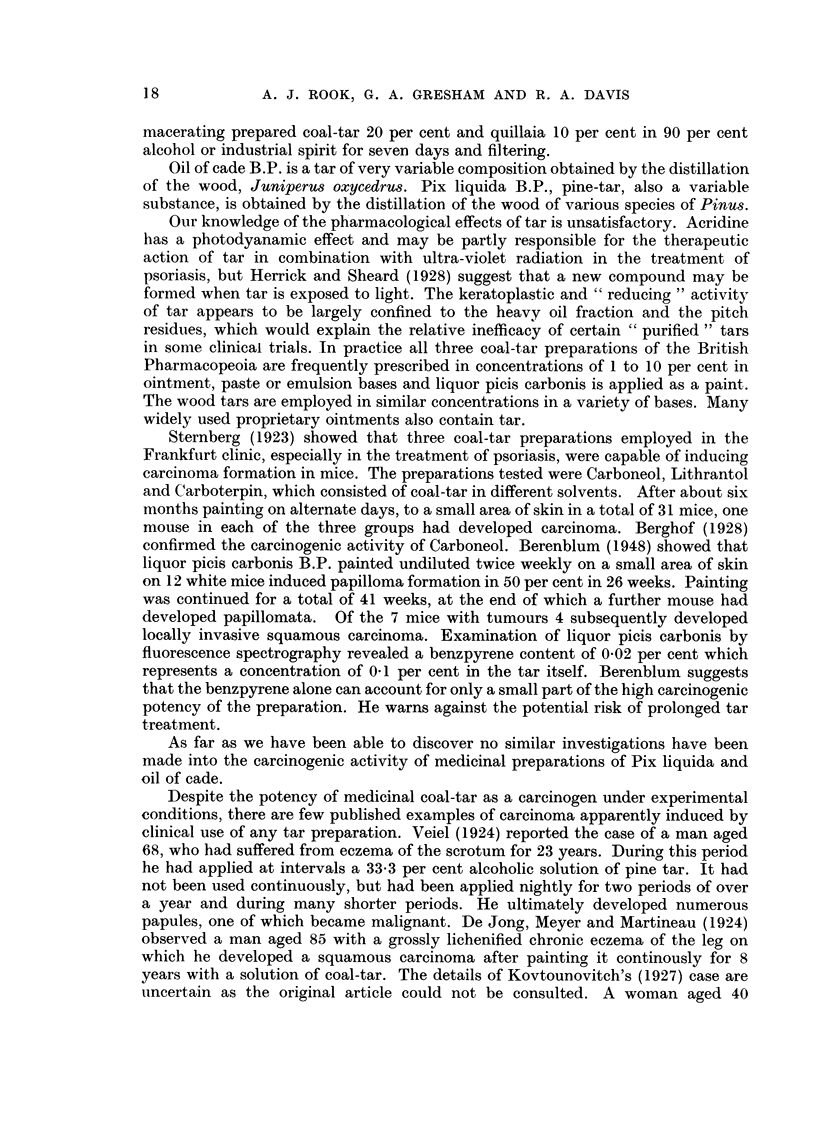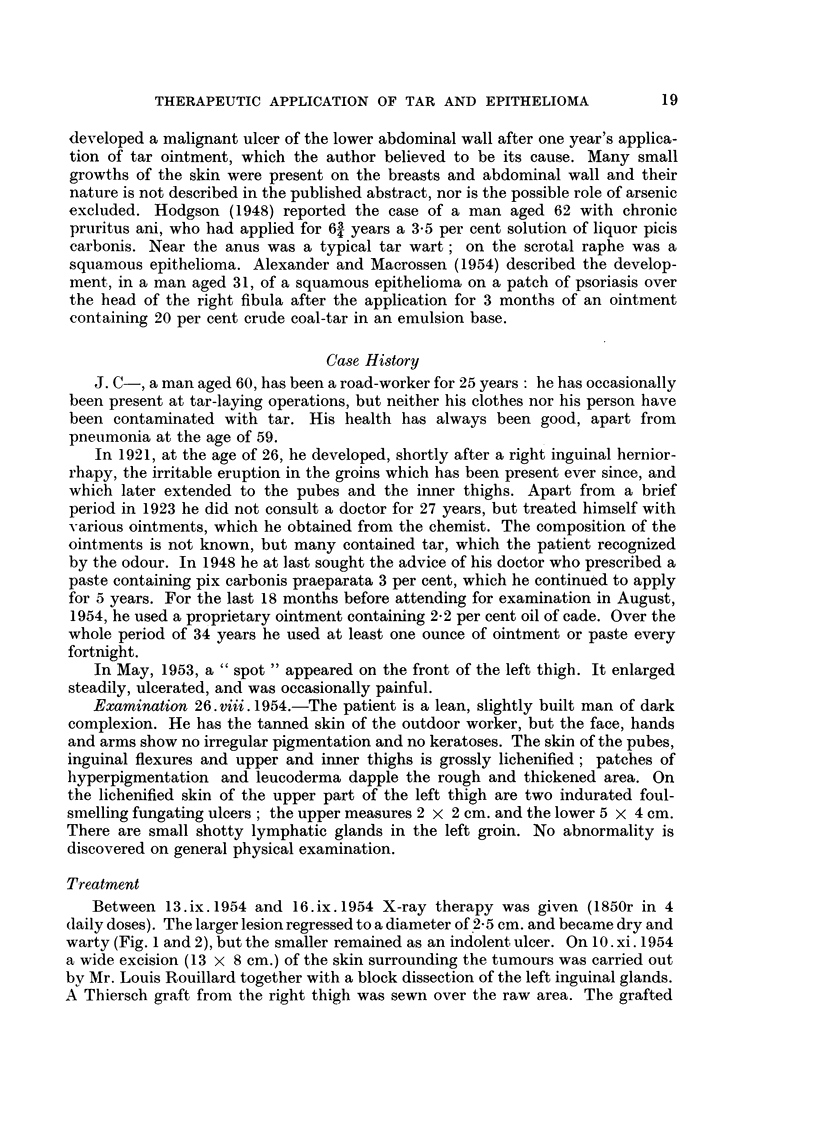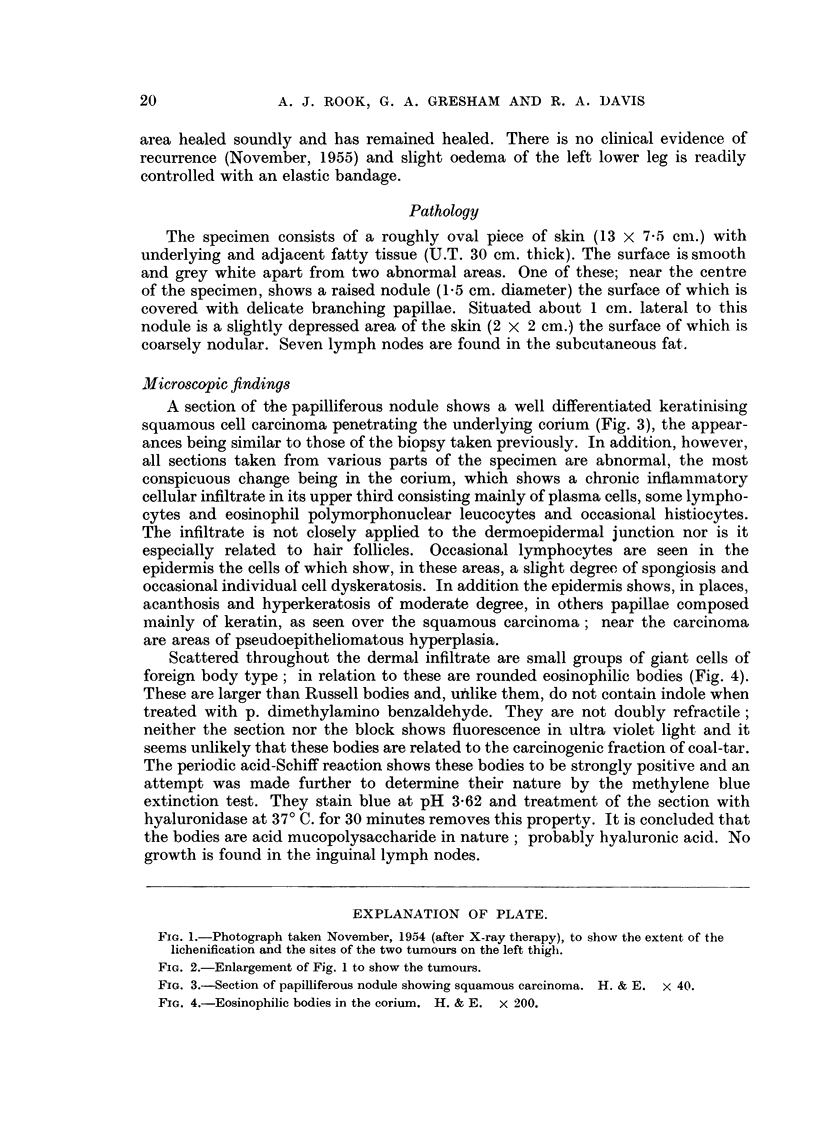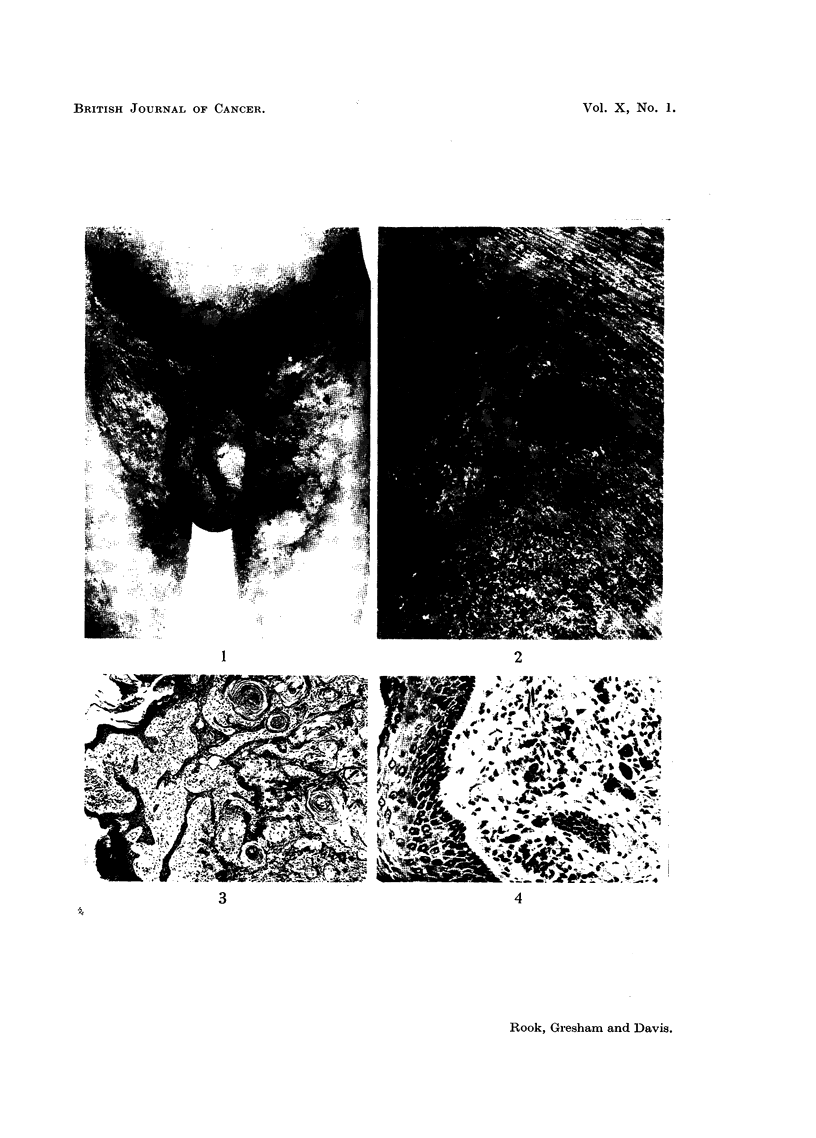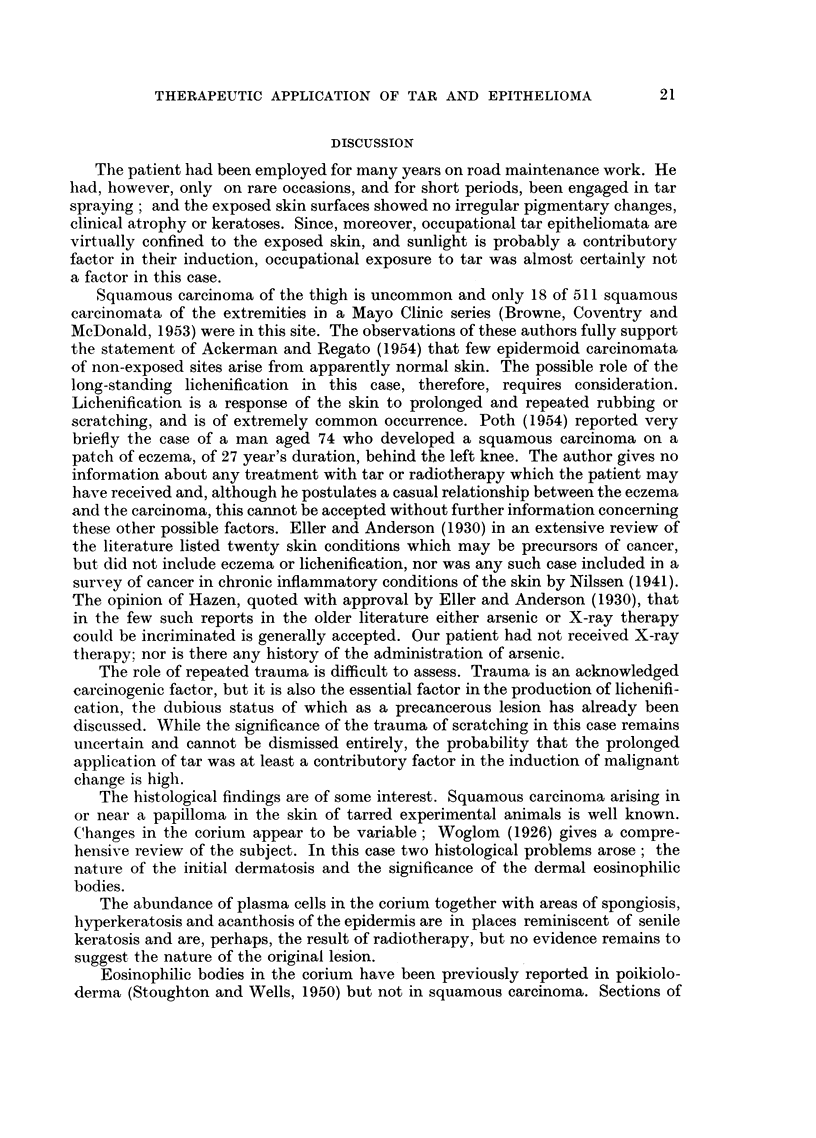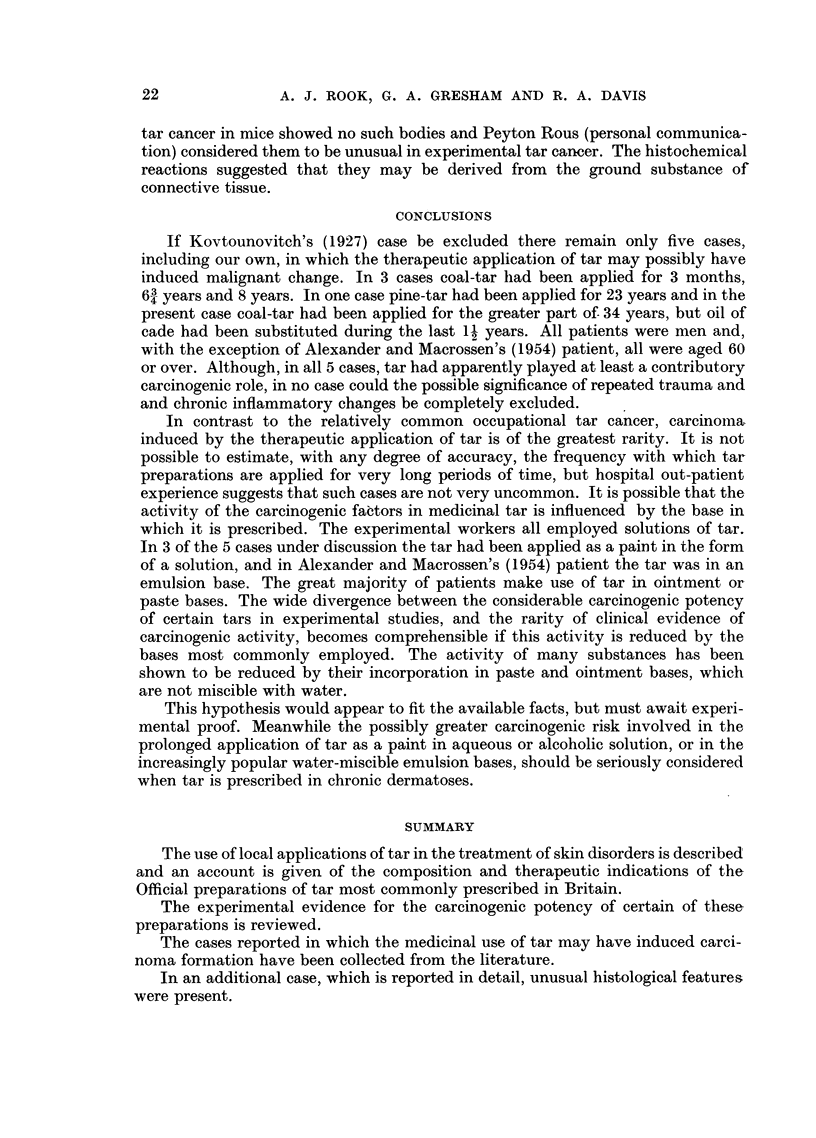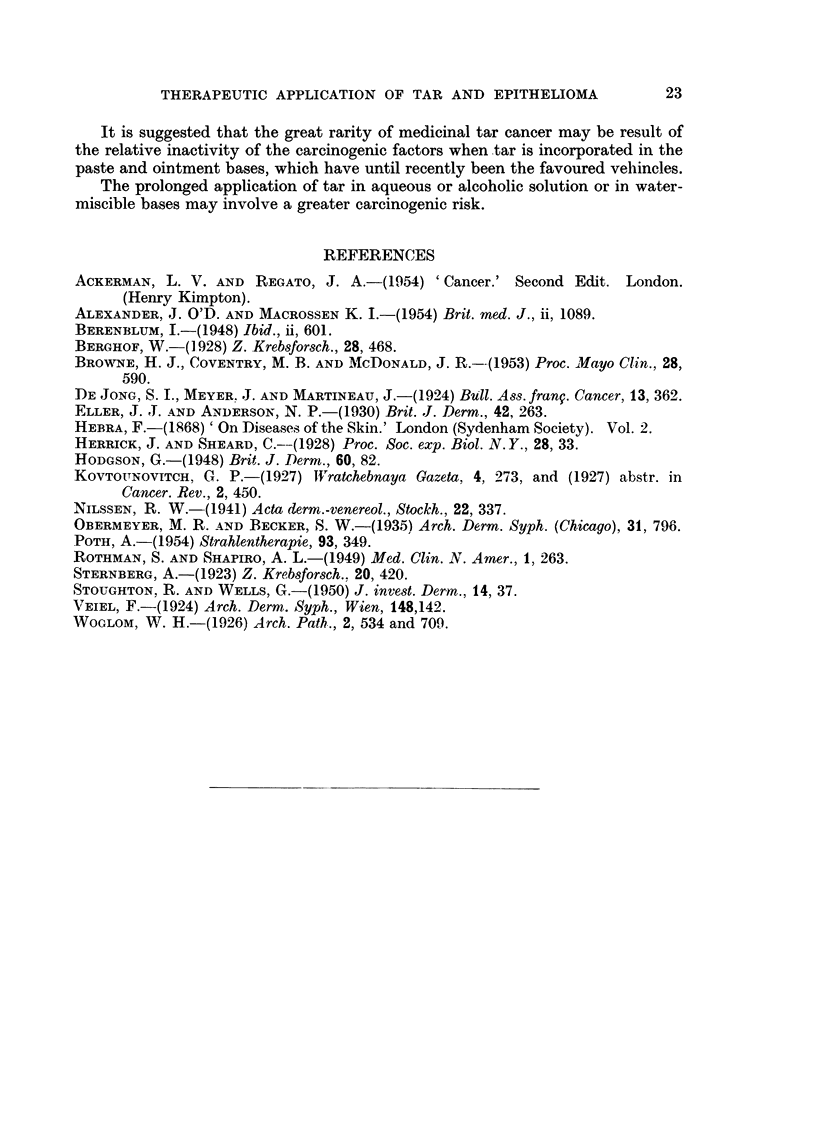# Squamous Epithelioma possibly Induced by the Therapeutic Application of Tar

**DOI:** 10.1038/bjc.1956.2

**Published:** 1956-03

**Authors:** A. J. Rook, G. A. Gresham, R. A. Davis

## Abstract

**Images:**


### SQUAMOUS EPITHELIOMA POSSIBLY INDUCED BY THE THERAPEUTIC APPLICATION OF TAR

A. J. ROOK, G. A. GRESHAM AND R. A. DAVIS

From the Department of Dermatology, Addenbrooke's Hospital, and the Department of Pathology, University of Cambridge

Received for publication January 18, 1956

THE employment of tar in dermatology has a long history. According to Hebra (1868), Theophrastus, Dioscorides and Pliny advocated its use in the treatment of diseases of the skin. For many centuries this treatment fell into disfavour, partly on account of the general acceptance of Galen's " acrimonia sanguinis " theory of the causation of skin disease which discouraged the use of local applications. Hebra gives credit for the re-introduction of tar in dermatology to Bateman and Wilkinson in England, to Rayer, Cazenave and others in France, and to Hertwig, Krieg, Otto and others in Germany and Austria. Until the second half of the nineteenth century only the wood tars were generally available. Contemporary text-books give evidence of their increasing popularity and Pix liquida appeared in the 1867 edition of the British Pharmacopoeia. After the introduction of coal-tar, which was in use for some years before it gained admission to the Pharmacopoeia in 1898, it slowly replaced the wood-tars, but these have not been entirely superseded, and both wood- and coal-tars still form an indispensable part of the dermatologists' armamentarium.

Tars are employed as anti-pruritics and " reducing agents ". The latter term was coined by Unna and has been defined as " agents which reduce epithelial proliferation, hyperkeratosis and cutaneous infiltrates and normalize faulty keratinisation " (Rothman and Shapiro 1949). Tars are of particular value in the treatment of eczema, lichenification and psoriasis ; since these conditions may persist or may recur at frequent intervals for many years it is not unusual for the patient, on medical advice or on his own initiative, to continue for very long periods treatment which provides some symptomatic relief.

The coal-tars are in general more often prescribed than the wood and bituminous tars. The official preparations of coal-tar in Britain are : pix carbonis B.P., pix carbonis praeparata B.P., liquor picis carbonis B.P. Pix carbonis is crude commercial coal-tar, a complex mixture of variable composition, containing predominantly the aromatic hydrocarbons, such as benzol, toluol, naphthalene and anthracene and phenolic substances such as phenol, cresol and various polyhydroxyphenols (Obermeyer and Becker, 1935). By dry distillation four fractions can be distinguished ; the light oils (benzol, toluol, xylene and pyridine) medium oils (naphtha, heavy benzene, crude phenol and cresols), and heavy oils (naphthalene, cresols, quinoline, etc.) and the anthracene oils (flourescent green oil, containing acridine and its derivatives). The remainder consists of pitch. Pix carbonis praeparata is commercial coal-tar heated at 50° C. for one hour, in which process certain of the lighter oil fractions are lost. Liquor picis carbonis is made by macerating prepared coal-tar 20 per cent and quillaia 10 per cent in 90 per cent alcohol or industrial spirit for seven days and filtering.

Oil of cade B.P. is a tar of very variable composition obtained by the distillation of the wood, *Juniperus oxycedrus*. Pix liquida B.P., pine-tar, also a variable substance, is obtained by the distillation of the wood of various species of *Pinus*.

Our knowledge of the pharmacological effects of tar is unsatisfactory. Acridine has a photodyanamic effect and may be partly responsible for the therapeutic action of tar in combination with ultra-violet radiation in the treatment of psoriasis, but Herrick and Sheard (1928) suggest that a new compound may be formed when tar is exposed to light. The keratoplastic and " reducing " activity of tar appears to be largely confined to the heavy oil fraction and the pitch residues, which would explain the relative inefficacy of certain " purified " tars in some clinical trials. In practice all three coal-tar preparations of the British Pharmacopeoia are frequently prescribed in concentrations of 1 to 10 per cent in ointment, paste or emulsion bases and liquor picis carbonis is applied as a paint. The wood tars are employed in similar concentrations in a variety of bases. Many widely used proprietary ointments also contain tar.

Sternberg (1923) showed that three coal-tar preparations employed in the Frankfurt clinic, especially in the treatment of psoriasis, were capable of inducing carcinoma formation in mice. The preparations tested were Carboneol, Lithrantol and Carboterpin, which consisted of coal-tar in different solvents. After about six months painting on alternate days, to a small area of skin in a total of 31 mice, one mouse in each of the three groups had developed carcinoma. Berghof (1928) confirmed the carcinogenic activity of Carboneol. Berenblum (1948) showed that liquor picis carbonis B.P. painted undiluted twice weekly on a small area of skin on 12 white mice induced papilloma formation in 50 per cent in 26 weeks. Painting was continued for a total of 41 weeks, at the end of which a further mouse had developed papillomata. Of the 7 mice with tumours 4 subsequently developed locally invasive squamous carcinoma. Examination of liquor picis carbonis by fluorescence spectrography revealed a benzpyrene content of 0·02 per cent which represents a concentration of 0·1 per cent in the tar itself. Berenblum suggests that the benzpyrene alone can account for only a small part of the high carcinogenic potency of the preparation. He warns against the potential risk of prolonged tar treatment.

As far as we have been able to discover no similar investigations have been made into the carcinogenic activity of medicinal preparations of Pix liquida and oil of cade.

Despite the potency of medicinal coal-tar as a carcinogen under experimental conditions, there are few published examples of carcinoma apparently induced by clinical use of any tar preparation. Veiel (1924) reported the case of a man aged 68, who had suffered from eczema of the scrotum for 23 years. During this period he had applied at intervals a 33·3 per cent alcoholic solution of pine tar. It had not been used continuously, but had been applied nightly for two periods of over a year and during many shorter periods. He ultimately developed numerous papules, one of which became malignant. De Jong, Meyer and Martineau (1924) observed a man aged 85 with a grossly lichenified chronic eczema of the leg on which he developed a squamous carcinoma after painting it continously for 8 years with a solution of coal-tar. The details of Kovtounovitch's (1927) case are uncertain as the original article could not be consulted. A woman aged 40

developed a malignant ulcer of the lower abdominal wall after one year's application of tar ointment, which the author believed to be its cause. Many small growths of the skin were present on the breasts and abdominal wall and their nature is not described in the published abstract, nor is the possible role of arsenic excluded. Hodgson (1948) reported the case of a man aged 62 with chronic pruritus ani, who had applied for 6¾ years a 3·5 per cent solution of liquor picis carbonis. Near the anus was a typical tar wart; on the scrotal raphe was a squamous epithelioma. Alexander and Macrossen (1954) described the development, in a man aged 31, of a squamous epithelioma on a patch of psoriasis over the head of the right fibula after the application for 3 months of an ointment containing 20 per cent crude coal-tar in an emulsion base.

### *Case History*

J. C—, a man aged 60, has been a road-worker for 25 years: he has occasionally been present at tar-laying operations, but neither his clothes nor his person have been contaminated with tar. His health has always been good, apart from pneumonia at the age of 59.

In 1921, at the age of 26, he developed, shortly after a right inguinal herniorrhapy, the irritable eruption in the groins which has been present ever since, and which later extended to the pubes and the inner thighs. Apart from a brief period in 1923 he did not consult a doctor for 27 years, but treated himself with various ointments, which he obtained from the chemist. The composition of the ointments is not known, but many contained tar, which the patient recognized by the odour. In 1948 he at last sought the advice of his doctor who prescribed a paste containing pix carbonis praeparata 3 per cent, which he continued to apply for 5 years. For the last 18 months before attending for examination in August, 1954, he used a proprietary ointment containing 2·2 per cent oil of cade. Over the whole period of 34 years he used at least one ounce of ointment or paste every fortnight.

In May, 1953, a " spot " appeared on the front of the left thigh. It enlarged steadily, ulcerated, and was occasionally painful.

*Examination* 26.*viii*.1954.—The patient is a lean, slightly built man of dark complexion. He has the tanned skin of the outdoor worker, but the face, hands and arms show no irregular pigmentation and no keratoses. The skin of the pubes, inguinal flexures and upper and inner thighs is grossly lichenified; patches of hyperpigmentation and leucoderma dapple the rough and thickened area. On the lichenified skin of the upper part of the left thigh are two indurated foul-smelling fungating ulcers; the upper measures 2 × 2 cm. and the lower 5 × 4 cm. There are small shotty lymphatic glands in the left groin. No abnormality is discovered on general physical examination.

### *Treatment*

Between 13.ix.1954 and 16.ix.1954 X-ray therapy was given (1850r in 4 daily doses). The larger lesion regressed to a diameter of 2·5 cm. and became dry and warty (Fig. 1 and 2), but the smaller remained as an indolent ulcer. On 10.xi.1954 a wide excision (13 × 8 cm.) of the skin surrounding the tumours was carried out by Mr. Louis Rouillard together with a block dissection of the left inguinal glands. A Thiersch graft from the right thigh was sewn over the raw area. The grafted area healed soundly and has remained healed. There is no clinical evidence of recurrence (November, 1955) and slight oedema of the left lower leg is readily controlled with an elastic bandage.

### *Pathology*

The specimen consists of a roughly oval piece of skin (13 × 7·5 cm.) with underlying and adjacent fatty tissue (U.T. 30 cm. thick). The surface is smooth and grey white apart from two abnormal areas. One of these; near the centre of the specimen, shows a raised nodule (1·5 cm. diameter) the surface of which is covered with delicate branching papillae. Situated about 1 cm. lateral to this nodule is a slightly depressed area of the skin (2 × 2 cm.) the surface of which is coarsely nodular. Seven lymph nodes are found in the subcutaneous fat.

#### *Microscopic findings*

A section of the papilliferous nodule shows a well differentiated keratinising squamous cell carcinoma penetrating the underlying corium (Fig. 3), the appearances being similar to those of the biopsy taken previously. In addition, however, all sections taken from various parts of the specimen are abnormal, the most conspicuous change being in the corium, which shows a chronic inflammatory cellular infiltrate in its upper third consisting mainly of plasma cells, some lymphocytes and eosinophil polymorphonuclear leucocytes and occasional histiocytes. The infiltrate is not closely applied to the dermoepidermal junction nor is it especially related to hair follicles. Occasional lymphocytes are seen in the epidermis the cells of which show, in these areas, a slight degree of spongiosis and occasional individual cell dyskeratosis. In addition the epidermis shows, in places, acanthosis and hyperkeratosis of moderate degree, in others papillae composed mainly of keratin, as seen over the squamous carcinoma ; near the carcinoma are areas of pseudoepitheliomatous hyperplasia.

Scattered throughout the dermal infiltrate are small groups of giant cells of foreign body type ; in relation to these are rounded eosinophilic bodies (Fig. 4). These are larger than Russell bodies and, unlike them, do not contain indole when treated with p. dimethylamino benzaldehyde. They are not doubly refractile ; neither the section nor the block shows fluorescence in ultra violet light and it seems unlikely that these bodies are related to the carcinogenic fraction of coal-tar. The periodic acid-Schiff reaction shows these bodies to be strongly positive and an attempt was made further to determine their nature by the methylene blue extinction test. They stain blue at pH 3·62 and treatment of the section with hyaluronidase at 37° C. for 30 minutes removes this property. It is concluded that the bodies are acid mucopolysaccharide in nature ; probably hyaluronic acid. No growth is found in the inguinal lymph nodes.

---

EXPLANATION OF PLATE.

FIG. 1.—Photograph taken November, 1954 (after X-ray therapy), to show the extent of the lichenification and the sites of the two tumours on the left thigh.

FIG. 2.—Enlargement of Fig. 1 to show the tumours.

FIG. 3.—Section of papilliferous nodule showing squamous carcinoma. H. & E. × 40.

FIG. 4.—Eosinophilic bodies in the corium. H. & E. × 200.

1

2

3

4

### DISCUSSION

The patient had been employed for many years on road maintenance work. He had, however, only on rare occasions, and for short periods, been engaged in tar spraying ; and the exposed skin surfaces showed no irregular pigmentary changes, clinical atrophy or keratoses. Since, moreover, occupational tar epitheliomata are virtually confined to the exposed skin, and sunlight is probably a contributory factor in their induction, occupational exposure to tar was almost certainly not a factor in this case.

Squamous carcinoma of the thigh is uncommon and only 18 of 511 squamous carcinomata of the extremities in a Mayo Clinic series (Browne, Coventry and McDonald, 1953) were in this site. The observations of these authors fully support the statement of Ackerman and Regato (1954) that few epidermoid carcinomata of non-exposed sites arise from apparently normal skin. The possible role of the long-standing lichenification in this case, therefore, requires consideration. Lichenification is a response of the skin to prolonged and repeated rubbing or scratching, and is of extremely common occurrence. Poth (1954) reported very briefly the case of a man aged 74 who developed a squamous carcinoma on a patch of eczema, of 27 year's duration, behind the left knee. The author gives no information about any treatment with tar or radiotherapy which the patient may have received and, although he postulates a casual relationship between the eczema and the carcinoma, this cannot be accepted without further information concerning these other possible factors. Eller and Anderson (1930) in an extensive review of the literature listed twenty skin conditions which may be precursors of cancer, but did not include eczema or lichenification, nor was any such case included in a survey of cancer in chronic inflammatory conditions of the skin by Nilssen (1941). The opinion of Hazen, quoted with approval by Eller and Anderson (1930), that in the few such reports in the older literature either arsenic or X-ray therapy could be incriminated is generally accepted. Our patient had not received X-ray therapy; nor is there any history of the administration of arsenic.

The role of repeated trauma is difficult to assess. Trauma is an acknowledged carcinogenic factor, but it is also the essential factor in the production of lichenification, the dubious status of which as a precancerous lesion has already been discussed. While the significance of the trauma of scratching in this case remains uncertain and cannot be dismissed entirely, the probability that the prolonged application of tar was at least a contributory factor in the induction of malignant change is high.

The histological findings are of some interest. Squamous carcinoma arising in or near a papilloma in the skin of tarred experimental animals is well known. Changes in the corium appear to be variable ; Woglom (1926) gives a comprehensive review of the subject. In this case two histological problems arose ; the nature of the initial dermatosis and the significance of the dermal eosinophilic bodies.

The abundance of plasma cells in the corium together with areas of spongiosis, hyperkeratosis and acanthosis of the epidermis are in places reminiscent of senile keratosis and are, perhaps, the result of radiotherapy, but no evidence remains to suggest the nature of the original lesion.

Eosinophilic bodies in the corium have been previously reported in poikiloderma (Stoughton and Wells, 1950) but not in squamous carcinoma. Sections of tar cancer in mice showed no such bodies and Peyton Rous (personal communication) considered them to be unusual in experimental tar cancer. The histochemical reactions suggested that they may be derived from the ground substance of connective tissue.

### CONCLUSIONS

If Kovtounovitch's (1927) case be excluded there remain only five cases, including our own, in which the therapeutic application of tar may possibly have induced malignant change. In 3 cases coal-tar had been applied for 3 months, $6\frac{3}{4}$ years and 8 years. In one case pine-tar had been applied for 23 years and in the present case coal-tar had been applied for the greater part of 34 years, but oil of cade had been substituted during the last $1\frac{1}{2}$ years. All patients were men and, with the exception of Alexander and Macrossen's (1954) patient, all were aged 60 or over. Although, in all 5 cases, tar had apparently played at least a contributory carcinogenic role, in no case could the possible significance of repeated trauma and and chronic inflammatory changes be completely excluded.

In contrast to the relatively common occupational tar cancer, carcinoma induced by the therapeutic application of tar is of the greatest rarity. It is not possible to estimate, with any degree of accuracy, the frequency with which tar preparations are applied for very long periods of time, but hospital out-patient experience suggests that such cases are not very uncommon. It is possible that the activity of the carcinogenic factors in medicinal tar is influenced by the base in which it is prescribed. The experimental workers all employed solutions of tar. In 3 of the 5 cases under discussion the tar had been applied as a paint in the form of a solution, and in Alexander and Macrossen's (1954) patient the tar was in an emulsion base. The great majority of patients make use of tar in ointment or paste bases. The wide divergence between the considerable carcinogenic potency of certain tars in experimental studies, and the rarity of clinical evidence of carcinogenic activity, becomes comprehensible if this activity is reduced by the bases most commonly employed. The activity of many substances has been shown to be reduced by their incorporation in paste and ointment bases, which are not miscible with water.

This hypothesis would appear to fit the available facts, but must await experimental proof. Meanwhile the possibly greater carcinogenic risk involved in the prolonged application of tar as a paint in aqueous or alcoholic solution, or in the increasingly popular water-miscible emulsion bases, should be seriously considered when tar is prescribed in chronic dermatoses.

### SUMMARY

The use of local applications of tar in the treatment of skin disorders is described and an account is given of the composition and therapeutic indications of the Official preparations of tar most commonly prescribed in Britain.

The experimental evidence for the carcinogenic potency of certain of these preparations is reviewed.

The cases reported in which the medicinal use of tar may have induced carcinoma formation have been collected from the literature.

In an additional case, which is reported in detail, unusual histological features were present.

It is suggested that the great rarity of medicinal tar cancer may be result of the relative inactivity of the carcinogenic factors when tar is incorporated in the paste and ointment bases, which have until recently been the favoured vehincles.

The prolonged application of tar in aqueous or alcoholic solution or in water-miscible bases may involve a greater carcinogenic risk.

### REFERENCES

ACKERMAN, L. V. AND REGATO, J. A.—(1954) 'Cancer.' Second Edit. London. (Henry Kimpton).

ALEXANDER, J. O'D. AND MACROSSEN K. I.—(1954) *Brit. med. J.*, ii, 1089.

BERENBLUM, I.—(1948) *Ibid.*, ii, 601.

BERGHOF, W.—(1928) *Z. Krebsforsch.*, **28**, 468.

BROWNE, H. J., COVENTRY, M. B. AND McDONALD, J. R.—(1953) *Proc. Mayo Clin.*, **28**, 590.

DE JONG, S. I., MEYER, J. AND MARTINEAU, J.—(1924) *Bull. Ass. franç. Cancer*, **13**, 362.

ELLER, J. J. AND ANDERSON, N. P.—(1930) *Brit. J. Derm.*, **42**, 263.

HEBRA, F.—(1868) 'On Diseases of the Skin.' London (Sydenham Society). Vol. 2.

HERRICK, J. AND SHEARD, C.—(1928) *Proc. Soc. exp. Biol. N.Y.*, **28**, 33.

HODGSON, G.—(1948) *Brit. J. Derm.*, **60**, 82.

KOVTOUNOVITCH, G. P.—(1927) *Wratchebnaya Gazeta*, **4**, 273, and (1927) abstr. in *Cancer. Rev.*, **2**, 450.

NILSSEN, R. W.—(1941) *Acta derm.-venereol., Stockh.*, **22**, 337.

OBERMEYER, M. R. AND BECKER, S. W.—(1935) *Arch. Derm. Syph. (Chicago)*, **31**, 796.

POTH, A.—(1954) *Strahlentherapie*, **93**, 349.

ROTHMAN, S. AND SHAPIRO, A. L.—(1949) *Med. Clin. N. Amer.*, **1**, 263.

STERNBERG, A.—(1923) *Z. Krebsforsch.*, **20**, 420.

STOUGHTON, R. AND WELLS, G.—(1950) *J. invest. Derm.*, **14**, 37.

VEIEL, F.—(1924) *Arch. Derm. Syph., Wien*, **148**,142.

WOGLOM, W. H.—(1926) *Arch. Path.*, **2**, 534 and 709.